# LILRB2-containing small extracellular vesicles from glioblastoma promote tumor progression by promoting the formation and expansion of myeloid-derived suppressor cells

**DOI:** 10.1007/s00262-023-03395-6

**Published:** 2023-02-28

**Authors:** Peitao Wu, Yuhang Guo, Li Xiao, Jiaqi Yuan, Chao Tang, Jun Dong, Zhiyuan Qian

**Affiliations:** 1 Department of Neurosurgery, The Second Affiliated Hospital of Soochow University, Soochow, 215000 People’s Republic of China; 2 Department of Neurosurgery, The First Affiliated Hospital of Wenzhou Medical University, Nanbaixiang, Wenzhou, China; 3 Department of Neurosurgery, Huashan Hospital, Shanghai, China

**Keywords:** Glioblastoma, LILRB2, Small extracellular vesicle (sEVs), MDSCs

## Abstract

**Background:**

Leukocyte immunoglobulin-like receptor subfamily B2 (LILRB2) was reported to be an inhibitory molecule with suppressive functions. sEVs mediate communication between cancer cells and other cells. However, the existence of LILRB2 on sEVs in circulation and the function of sEVs-LILRB2 are still unknown. This study aims to investigate the role of LILRB2 in GBM and determine how LILRB2 in sEVs regulates tumor immunity.

**Methods:**

LILRB2 expression in normal brain and GBM tissues was detected by immunohistochemistry, and the effect of LILRB2 on prognosis was evaluated in an orthotopic brain tumor model. Next, a subcutaneous tumor model was constructed to evaluate the function of pirb in vivo. The immune cells in the tumor sites and spleen were detected by immunofluorescence staining and flow cytometry. Then, the presence of pirb in sEVs was confirmed by WB. The percentage of immune cells after incubation with sEVs from GL261 (GL261-sEVs) or sEVs from GL261-pirb^+^ (GL261-sEVs-pirb) was detected by flow cytometry. Then, the effect of pirb on sEVs was evaluated by a tumor-killing assay and proliferation assay. Finally, subcutaneous tumor models were constructed to evaluate the function of pirb on sEVs.

**Results:**

LILRB2 was overexpressed in human GBM tissue and was closely related to an immunosuppressive TME in GBM. Then, a protumor ability of LILRB2 was observed in subcutaneous tumor models, which was related to lower CD8 + T cells and higher MDSCs (myeloid-derived suppressor cells) in the tumor and spleen compared to those of the control group. Next, we found that pirb on sEVs (sEVs-pirb) inhibits the function of CD8 + T cells by promoting the formation and expansion of MDSCs. Furthermore, the protumor function of sEVs-pirb was demonstrated in subcutaneous tumor models.

**Conclusion:**

We discovered that LILRB2/pirb can be transmitted between GBM cells via sEVs and that pirb on sEVs induces the formation and expansion of MDSCs. The induced MDSCs facilitate the formation of an immunosuppressive TME.

**Graphical Abstract:**

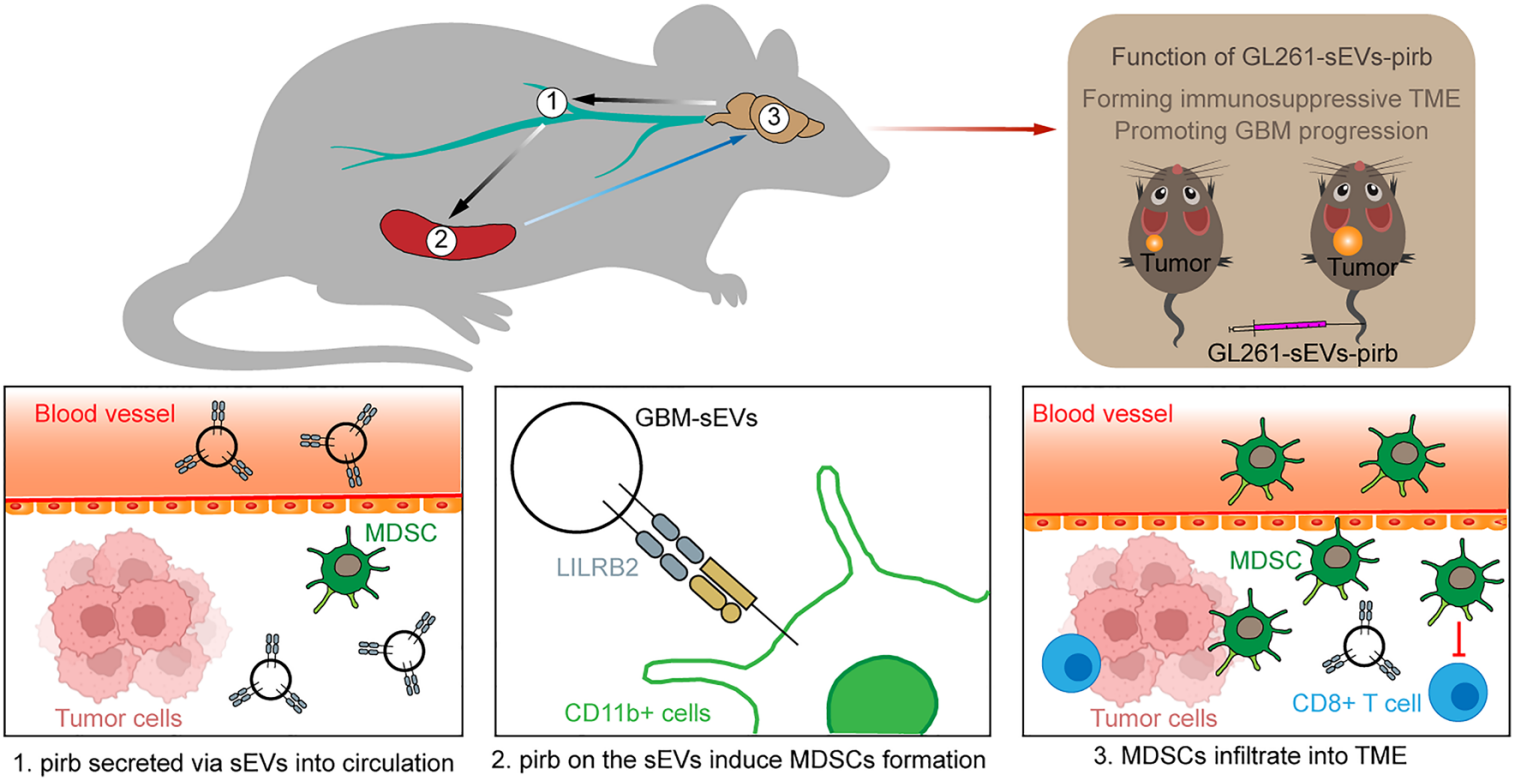

**Supplementary Information:**

The online version contains supplementary material available at 10.1007/s00262-023-03395-6.

## Background

Glioblastoma (GBM) is a lethal disease of the central nervous system [1]. Although surgery and radiochemotherapy have been used as first-line therapies in the clinic, the overall survival is still less than 14 months [2]. Multiple types of treatments have been explored to improve the prognosis of GBM patients over the past decade, especially immunotherapy [3, 4]. Immunotherapy with checkpoint inhibitors is becoming a promising treatment in some tumors by reversing the suppression of CD8 + T cells [5, 6]. However, the benefit of immune checkpoint blockade (ICB) therapy for GBM is still limited because of the immunosuppressive tumor microenvironment (TME) [7]. Although studies have shown that the outcome of immunotherapy can be enhanced by reverting the immunosuppressive TME, more effort is needed to determine the formation mechanism of the immunosuppressive TME to improve the prognosis of GBM patients.

The immunosuppressive TEM of GBM mainly consists of brain-resident microglia, tumor-associated macrophages (TAMs), myeloid-derived suppressor cells (MDSCs) and regulatory T (Treg) cells [8]. Although the formation mechanisms of this immunosuppressive TME are not completely understood, studies have shown that tumor-induced MDSCs play a pivotal role in this process [9, 10]. MDSCs are immature myeloid cells derived from bone morrow that accumulate in lymphoid organs, tumor sites and peripheral blood circulation [11] and characteristically express CD11b + Gr-1 + in mice and CD11b + CD33 + in humans [12]. These cells exert their immunosuppressive function by releasing immunomodulatory cytokines and inhibiting T cells [13]. Evidence suggests that MDSCs can differentiate into TAMs in tumor sites, resulting in an elevation of IL-10 and inhibition of T-cell function [14]. Another study showed that MDSCs promote the formation and expansion of Tregs in tumor model mice [15]. Furthermore, the level of circulating MDSCs was significantly increased in GBM patients compared with healthy donors, and the average level of MDSCs also increased significantly with the tumor grade. Moreover, the immunotherapy response and prognosis of GBM patients can be significantly improved by reducing MDSCs in the TME [16]. We speculate that the increased MDSCs in the circulation of tumor patients may be related to the level of infiltrated MDSCs in the TME.

LILRB2 (leukocyte immunoglobulin-like receptor subfamily B2) is also named immunoglobulin-like transcript 4 (ILT4), and PirB is the only orthologous gene in mice. Previous research has shown that LILRB2 is mainly expressed in myeloid cells [17]. Recently, studies have shown that LILRB2 was upregulated in lung adenocarcinoma tissues and correlated with reduced CD8 + T-cell infiltration, advanced disease progression and poor prognosis [18]. Another study showed that LILRB2 inhibits the function of CD8 + T cells, while downregulating the expression of LILRB2 impaired the recruitment of tumor-associated macrophages [19]. However, the function of pirb/LILRB2 in GBM remains to be elucidated.

Recently, small extracellular vesicles (sEVs) have attracted increased attention as a kind of novel cell communication that exerts their function by carrying bioactive molecules [20]. Importantly, tumor cell-derived sEVs play a pivotal role in the progression of tumors. The secreted sEVs transport TGFβ from cancer cells to normal stromal fibroblasts, transforming fibroblasts into myofibroblasts [21], and a favorable tumor microenvironment was generated by carrying pyruvate kinase type M2 [22]. GBM cell-derived sEVs (GBM-sEVs) are an important way for GBM cells to communicate with other cells, including immune cells [23]. Studies have shown that tumor-derived sEVs participate in the induction of MDSCs [24], and MDSC-like properties can be induced in monocytes by exposing them to glioma cells [9]. However, the function of LILRB2 in GBM cells in MDSCs has yet to be determined.

In this study, we proposed that the secreted LILRB2 on GBM-sEVs suppresses the immune system via MDSCs and that the induced MDSCs in the circulation can infiltrate the TME, exerting their immunosuppressive function. Here, we speculate that LILRB2 will be a therapeutic target in the immunotherapy of GBM.

## Methods

### Bioinformatics analysis

The gene expression profile of LILRB2 in GBM and normal brain samples was obtained from GEPIA (http://gepia.cancer-pku.cn/), while the gene expression profile of LILRB2 in different grades (WHO II, WHO III and WHO IV) of glioma was obtained from CGGA (http://www.cgga.org.cn/).

### Patients and samples

GBM samples (*n* = 3) were collected from GBM patients without chemotherapy and radiotherapy. Normal brain tissues were collected from injured brain tissue. All these samples were collected in the Second Affiliated Hospital of Soochow University and approved by the ethics committee of the Second Affiliated Hospital of Soochow University.

### Immunohistochemistry (IHC) analysis

The collected tissues were fixed immediately in 4% paraformaldehyde solution and embedded in optimal cutting temperature (OCT) compound. The samples were cut into Sects. (10 μm) with a freezing microtome (Leica CM 1950; Leica Biosystem, Heidelberg, Germany). The sections were permeabilized with 0.1% Triton X-100 and blocked with 5% bovine serum albumin (BSA), followed by incubation with primary antibody and second antibody and staining with a DAB kit (Servicebio, China). The samples were imaged by DM6 microscopy (Leica, Germany). The antibodies used in IHC were as follows: ILT4 (1:200, Santa Cruz); secondary antibody: anti-mouse-HRP (1:1000, CST). The mean density (integrated option density/area) of the images was measure by Image Pro Plus software.

### Cell culture

The murine glioblastoma cell line GL261 and the human glioblastoma cell lines U87 and U251 were purchased from the Cell Bank of the Chinese Academy of Sciences (Shanghai, China). All these cell lines were cultured in Dulbecco’s modified Eagle’s medium (DMEM, Corning) containing 10% FBS (Gibco) and maintained at 37 °C in 5% CO_2_.

### Cell transfection and RNA interference

The pirb-overexpressing lentivirus, pirb-overexpressing lentivirus with red fluorescence protein and control lentivirus were purchased from Vigene Bioscience (Shandong, China). GL261 cells were plated in 6-well plates at a density of 5 × 10^5^ cells/well overnight, and the lentivirus was added to DMEM (MOI: 50). After 72 h, the infected cells were screened by puromycin (MCE, American) at a concentration of 1 μg/mL for 2 weeks. Thus, GL261 cells transfected with control lentivirus (GL261-LV-nc), GL261 cells transfected with pirb-overexpressing lentivirus (GL261-pirb^+^), GL261 cells transfected with pirb-overexpressing lentivirus with red fluorescence protein (GL261-pirb-RFP) were harvested.

Small interfering RNA (siRNA) targeting pirb was purchased from Bioneer (Korea). Lipofectamine RNAiMAX Reagents (Thermo Fisher, USA) were used in the transfection of siRNA in GL261 cells. The expression of pirb was detected by WB. Thus, GL261 cells transfected with siRNA of control (GL261-nc), and GL261 cells transfected with siRNA of pirb (GL261-pirb^−^) were harvested.

### Isolation of macro extracellular vesicles (MVs) and small extracellular vesicles (sEVs)

EV-depleted FBS was obtained by 18 h ultracentrifugation at 100,000 g [25]. After the cells were cultured in EV-depleted media for 72 h, conditioned media were collected and centrifuged at 400 × g for 10 min and 2000 × g for 15 min. The MVs were collected by centrifugation at 15,000 × g for 30 min, and then, the supernatant underwent ultracentrifugation at 100,000 × g for 75 min at 4 °C (Optima XPN-100 Ultracentrifuge, Beckman Coulter Life Sciences) to collect sEVs. The precipitate was resuspended in sterile PBS, and the suspension was ultracentrifuged again (100,000 g, 75 min, 4 °C) to collect the sEVs. Thus, the sEVs derived from GL261 cells (GL261-sEVs), the sEVs from GL261-pirb + (GL261-pirb-sEVs), the sEVs from GL261-pirb-RFP (GL261-pirb-RFP-sEVs) were harvested. The obtained sEVs were stored at − 80 °C.

Blood from control mice and tumor-bearing mice was collected, mixed with EDTA solution, and centrifuged at 500 g to collect the supernatant. Then, the supernatant was mixed with PBS of the same volume, and the sEVs were collected by ultracentrifugation (100,000 g, 75 min, 4 °C).

### Western blotting analysis

Cells and sEV samples were lysed with a Radio Immunoprecipitation Assay (RIPA, Beyotime Biotechnology, China) with phenylmethanesulfonyl fluoride (PMSF) (10 μL/1 mL) (Beyotime Biotechnology, China). The protein concentration was measured by a BCA assay (Beyotime Biotechnology, China). Then, the protein solution was mixed with loading buffer (Beyotime Biotechnology, China) and boiled at 95 °C for 10 min. Proteins were separated with SDS‒PAGE (Shanghai Epizyme Biomedical Technology, China) and transferred to a 0.45 μm polyvinylidenedifluoride (PVDF) membrane (Millipore, American). After that, the membranes were blocked with 5% (w/v) nonfat milk (Beyotime Biotechnology, China) for 90 min and incubated with primary antibody followed by secondary antibody. The primary antibodies used for immunoblotting were anti-pirb (1:1000, R&D Systems, MAB2754), anti-actin (1:1000, Invitrogen, MA5-15,739), anti-GM130 (1:1000, Abcam, ab52649), anti-CD63 (1:1000, Abcam, ab134045), anti-CD9 (1:1000, Abcam, ab92726), and anti-Alix (1:500, Santa Cruz, sc-53540). The secondary antibodies used for immunoblotting were anti-mouse IgG, HRP-linked antibody (1:2000, CST, 7076) and anti-rabbit, IgG, HRP-linked antibody (1:2000, CST, 7074).

### Animal model and administration

Four-week-old female nude mice and six-week-old C57BL/C mice were purchased from SLAC Laboratory Animal Company (Shanghai, China), and all animal experimental protocols were approved by the Animal Research Committee of the Soochow University (Approval No. SUDA20210708A03).

For analysis of the function of pirb in GBM, a subcutaneous tumor model was constructed with GL261 cells (3 × 10^6^, 100 μl) or GL261-pirb^+^ cells (3 × 10^6^, 100 μl) on the flanks of C57BL/C mice (*n* = 5). The tumor volumes were measured by calipers every four days and recorded. Four weeks later, these mice were killed, and the tumors were collected.

For survival analysis, an orthotopic brain tumor model of C57BL/C mice was constructed (*n* = 5). GL261 cells (1 × 10^5^, 1 μL) and GL261-pirb^+^ cells (1 × 10^5^, 1 μL) were injected into the right striatum of C57BL/C mice by a stereotactic instrument. Then, the survival time of these mice was recorded.

Subcutaneous tumor models were constructed on both sides of the C57BL/C mice. GL261 cells (3 × 10^6^, 100 μl) and GL261-pirb^+^ cells (3 × 10^6^, 100 μl) were injected into the left flanks and the right flanks of C57BL/C mice, respectively (*n* = 5). The tumor volumes were measured by calipers every four days and recorded for four weeks.

For analysis of the function of sEV-pirb-induced MDSCs in GBM, a subcutaneous tumor model was constructed with GL261 cells (3 × 10^6^, 100 μl) in the right flank of C57BL/C mice. MDSCs (1 × 10^6^) were injected intravenously when the tumor volume reached 100 mm^3^. Three days later, the tumors were harvested. The presence of MDSCs in tumors was detected by IF.

For analysis of the function of pirb-sEVs, GL261 cells (3 × 10^6^, 100 μl) were injected into the flanks of C57BL/C mice to construct subcutaneous xenograft tumor models. The tumor-bearing mice were divided into three groups when the tumor volume reached 100 mm^3^. The control group (*n* = 3) was treated with PBS (100 μL), while the other mice were treated with GL261-sEVs (1 × 10^11^, 100 μL) or GL261-pirb-sEVs (1 × 10^11^, 100 μL). The treatment was given every four days 3 times, and the tumor volume was measured with calipers. The mice were killed sixty days after the intervention, and the tumors were collected. Tumor volumes were calculated with the formula: tumor volume = L × W^2^/2.

### CCK8 assays

For the proliferation assay, GL261-nc, GL261-pirb^+^ and GL261-pirb^−^ cells were plated on a 96-well plate at a density of 8000 cells/well. Then, the cell viability was measured at 24, 48 and 72 h by a Cell Counting Kit-8 (CCK8; Dojindo) assay. For the cytotoxicity test, GL261 cells were plated on a 96-well plate at a density of 8000 cells/well. After 24 h, GW4869 (10 μM) was added to the DMEM culture medium. Cell viability was measured at 72 h according to the manufacturer’s protocol. The absorbance of each cell sample was detected at 450 nm using a plate reader (Bio-Rad, USA).

#### Cell cycle and cell apoptosis assays

GL261-nc cells, GL261-pirb^+^ cells and GL261-pirb^−^ cells were harvested and washed twice with PBS. For the cell cycle assay, the cells were stained with PE (Sigma, 25,535–16-4) at a concentration of 10 mg/mL according to the direction (*n* = 3). For the cell apoptosis assay, the cells were stained with an Annexin-V/FITC cell apoptosis kit (BD, America) according to the direction (*n* = 3). Then, the cells were collected and detected by flow cytometry.

#### Cell migration and invasion assay

For the migration assay, 5 × 10^3^ GL261-nc, GL261-pirb^+^ cells and GL261-pirb^−^ cells were seeded in a transwell plate (*n* = 3). After 24 h, the migrated cells were stained with crystal violet (Beyotime Biotechnology, China) and washed with PBS. Finally, the migrated cells were imaged with DM6 microscopy (Leica, Germany) and analyzed by ImageJ software. For the invasion assay, Matrigel (Corning, America) was mixed with PBS at a ratio of 1:8, and the mixture was added to the upper chamber of the transwell plate. Then, 5 × 10^3^ GL261-nc, GL261-pirb^+^ and GL261-pirb^−^ cells were seeded into the upper chamber (*n* = 3). After 24 h, the invaded cells were stained with crystal violet and washed with PBS. Finally, the invaded cells were imaged with DM6 microscopy (Leica, Germany) and analyzed by ImageJ software.

#### Immunofluorescence staining (IF)

Immune cells in tumor samples were detected by immunofluorescence staining. The samples were cut into pieces with a thickness of 10 μm and permeated with 0.1% Triton X-100 followed by blocking with 5% bovine serum albumin (BSA) for 1 h. Then, the sections were incubated with primary antibodies overnight at 4 °C, followed by incubation with the secondary antibodies for 1 h at room temperature. Finally, the nucleus was stained with 4',6-diamidino-2-phenylindole (DAPI, Beyotime Biotechnology, China). All these samples were observed under a Leica DM6B (Leica Microsystems, Germany). The primary antibodies used for immunofluorescence staining were as follows: CD4 (Biolegend, 1:200), CD8 (Biolegend, 1:200), CD11b (Biolegend, 1:200), CD33 (Biolegend, 1:200), and FOXP3 (Biolegend, 1:200).

#### Flow cytometry analysis

A single-cell suspension of spleen tissue was prepared as described previously. Briefly, the collected blood samples of mice were mixed with EDTA solution and centrifuged at 500 g. The collected cells were mixed with ACK buffer (A1049210, Thermo Fisher, USA). For splenocytes treated with sEVs, the cells were collected and washed twice with PBS. Next, the white blood cells were obtained by centrifugation. The single cells were stained with antibodies. The primary antibodies used in flow cytometry were as follows: CD3 (1:200, Biolegend), CD4 (1:200, Biolegend), CD8 (1:200, Biolegend), CD11b (1:200, Biolegend), Gr-1 (1:200, Biolegend), CD25 (1:200, Biolegend), and Foxp3 (1:200, Biolegend).

#### Characterization of sEVs

sEVs were characterized according to the guidelines of the International Society for Extracellular Vesicles [26].

Size Distribution and Particle Concentration: A nanoflow cytometer (N30 Nanoflow Analyzer; NanoFCM, Inc., Xiamen, China) was used to detect the diameter and concentration of sEVs from U251 cells (U251-sEVs), sEVs from U87 cells (U87-sEVs) and GL261-sEVs. For detection of the particle concentration, standard polystyrene nanoparticles (200 nm, concentration: 1.58 × 10^8^/mL, NanoFCM, Inc., Xiamen, China) were used to quantify sEVs. The sEV concentration was calculated according to the particle number ratio between the sEV samples and the standard nanoparticles. For size distribution measurement, standard silica nanoparticles (diameter: 68, 91, 113, 155 nm) were used to create a standard curve. The sEV samples were diluted and loaded into a nanoflow cytometer, and the size distribution was obtained.

Transmission electron microscopy (TEM): The morphology of U251-sEVs, U87-sEVs and GL261-sEVs was detected by TEM (Hitachi H-7650, Tokyo, Japan). Briefly, 10 μL of sEV solution (2 × 10^10^ particles/mL) was added onto a Formvar carbon-coated grid (300 mesh) and dried for 20 min. Then, the grid was washed with sterile PBS once and fixed with 1% (w/v) glutaraldehyde for 5 min. After that, the grid was washed with deionized (DI) water and stained with 2% (w/v) saturated aqueous uranyl oxalate for 5 min. Finally, the sEV-containing grid was dried for 10 min at room temperature, and the microstructure of the sEVs was imaged.

#### The binding assay of sEVs and myelocytes

GL261-sEVs were stained with DiI (10 μM), washed with PBS twice, and collected with ultracentrifugation. The DiI-labeled GL261-sEVs were incubated with splenocytes at a concentration of 1 × 10^9^ particles/mL. After 24 h, the splenocytes were fixed with 4% PFA for 30 min. Next, the splenocytes were incubated with antibody followed by incubation with DAPI. The images were observed by DM6 microscopy (Leica, Germany). The antibodies used were as follows: CD11b (1:200, Biolegend).

#### Uptake assay

sEVs (1 × 10^10^ particles/mL) were labeled with 10 μM DiO (Thermo Fisher, USA) at 37 °C for 20 min, washed with PBS twice and isolated by ultracentrifugation (100,000 g, 75 min, 4 °C). The DiO-labeled sEVs were added to the culture medium of spleen cells. Moreover, a parallel control group was established to eliminate the false positives induced by free DiO. In the parallel control group, 10 μM DiO in PBS was processed with the same procedures, the tube bottom was rinsed with PBS, and PBS was added to the culture medium. After 12 h, the cells were fixed with 4% paraformaldehyde for 20 min, permeabilized in 0.1% Triton-X 100 for 10 min, and stained with 4′,6-diamidino-2-phenylindole (DAPI, Beyotime Biotechnology, China). The stained cells were imaged with a confocal fluorescence microscope (Leica Microsystems, Wetzlar, Germany).

#### T-cell cytolytic assay

For determination CD8 + T-cell-mediated cytotoxicity, PBMCs from mice were obtained from the spleen as described before, and the obtained mouse PBMCs were diluted to 5 × 10^5^ cells/mL with Roswell Park Memorial Institute (RPMI) 1640 culture medium containing 10% FBS. For the cell coculture assay, T cells in PBMCs were stimulated with 10 μg/ml coated anti-CD3 and 2 μg/ml soluble anti-CD28 for 6 h. Then, the pretreated spleen cell suspension was mixed with GL261 cells (5 × 10^5^ cells/mL) at a ratio of 1:2. Next, 100 μL of the cell mixture was plated in a 96 × well plate. After that, GL261-nc-sEVs (1 × 10^9^ particles/mL) or GL261-pirb-sEVs (1 × 10^9^ particles/mL) were added to the cell mixture of 96-well plants. After 72 h, GL261 cell viability was detected by CCK8 assays.

#### Proliferation assay of CD8 + T cells

A single-cell suspension of spleen was prepared as described previously. Then, CD8 + T cells were isolated with a MojoSort™ Human CD8 T Cell Isolation Kit according to the manufacturer’s instructions. Then, the CD8 + T cells were stained with CFSE (10 μM, Thermo Fisher, USA) for 20 min, the staining was stopped with RPMI 1640, and the cells were washed twice with RPMI 1640 medium. The anti-CD3-pretreated CD8 + T cells were incubated with spleen cells, and GL261-sEVs or GL261-pirb-sEVs were added to the culture medium for 48 h. Finally, the treated CD8 + T cells were washed twice with PBS and detected by flow cytometry.

#### Isolation of induced MDSCs

The spleen was isolated from C57BL/C mice in a sterile context, and the isolated spleen was ground and filtered with a filter (). The single-cell suspension was centrifuged at 500 × g for 10 min. The supernatant was discarded, and the cells were resuspended with ACK lysis () for 5 min. Next, white cells were collected and centrifuged at 500 × g for 10 min. Then, the collected cells were incubated with GL261-pirb-sEVs (1 × 10^9^ particles/mL) for 72 h. The induced MDSCs were isolated with an EasySep™ Mouse MDSC (CD11b + Gr1 +) Isolation Kit (Stemcell, 19,867) and washed twice with PBS. The isolated MDSCs were intravenously injected into tumor-bearing mice.

#### MDSC migration assay

For analysis of the migration of the induced MDSCs, 5 × 10^4^ MDSCs were resuspended in 100 μL of low serum medium in the presence of GL261-LV-nc or GL261-pirb^+^ and plated in the upper chamber of a transwell plate. Then, 500 μL of DMEM was added to the lower chamber. After 24 h, the upper chamber was fixed with 4% paraformaldehyde for 15 min and then stained with 0.1% crystal violet for 10 min. The images were observed by DM6 microscopy (Leica, Germany).

#### Statistical analysis

Statistical analysis was performed with GraphPad Prism software (version 8.0.1). All results are expressed as the mean ± standard deviation. Statistical analysis was performed using Student's t test for comparisons between two groups. Differences were considered significant when *P < 0.05, **P < 0.01, ***P < 0.001, or ****P < 0.0001; ns indicates no statistical significance.

## Results

### High LILRB2/pirb expression correlated with poor prognosis

**Fig. 1:**
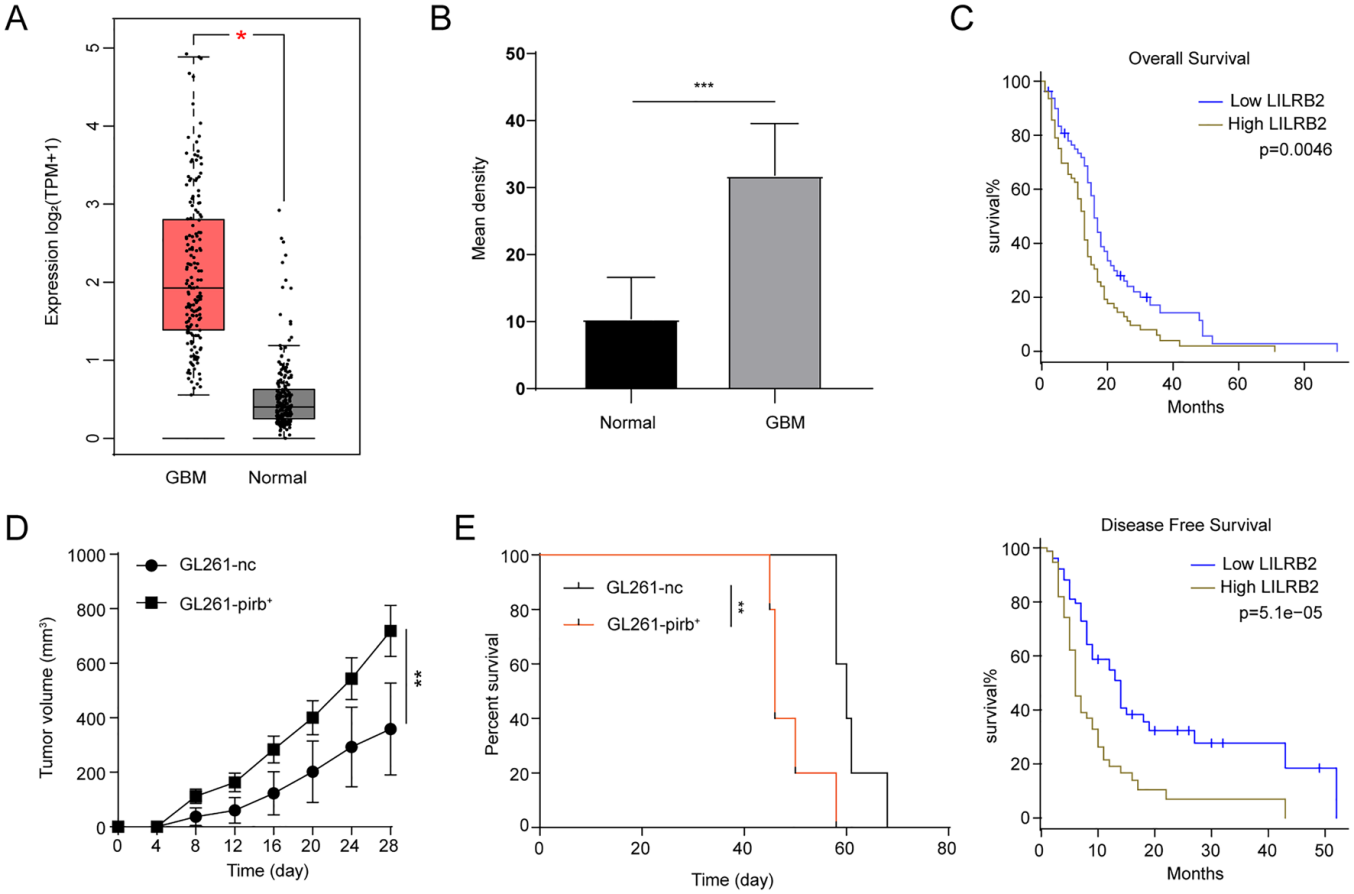
High LILRB2/pirb expression in GBM correlated with poor prognosis. **A** Gene expression profiling analysis of LILRB2 mRNA in GBM samples (n = 163) and normal brain tissues (*n* = 207). **B** The statistical analysis of the expression of LILRB2 in normal brain tissues (*n* = 8) and GBM tissues (*n* = 18) detected by IHC. **C** Kaplan–Meier analysis of overall survival and disease-free survival of GBM patients showing high LILRB2 expression (50% cutoff) and low LILRB2 expression (50% cutoff), the blue line refers to patients with low LILRB2, brow line refers to the patients with high LILRB2. **D** The tumor growth curve of GL261-nc (*n* = 5) and GL261-pirb (*n* = 5). **E** The survival times of the intracranial orthotopic tumor model constructed by GL261-nc (*n* = 5) or GL261-pirb cells (*n* = 5). *P < 0.05, ** P < 0.01

ICB therapy is becoming a promising treatment for tumors, and the outcome is closely related to the immune TME [27]. Evidence has shown that immunosuppressive proteins are pivotal in the regulation of the immune TME [28, 29]. We found that LILRB2 was highly expressed in human GBM tissues compared to normal human brain tissues (Fig. 1A) and positively related to pathological grade (Figure S1A). Subsequently, a greater number of LILRB2-positive cells was observed in GBM than in normal human brain tissues, as detected by IHC (Fig. 1B, Figure S1B). Next, we found that the expression level of LILRB2 in GBM was closely related to the prognosis of GBM patients. Higher expression of LILRB2 indicated shorter DFS and OS both in GBM patients (Fig. 1C) and high-grade glioma patients (Figure S1C), demonstrating that LILRB2 is an oncogene that indicates a dismal prognosis in GBM patients.

To explore the functional role of LILRB2 in GBM, we generated pirb-overexpressing GL261 cells (GL261-pirb), and the expression of pirb in GL261-pirb was confirmed by WB analysis (Figure S1D). Then, a subcutaneous tumor model was established in C57BL/C mice (Figure S1E). The mean GL261-nc tumor volumes reached 358.85 mm^3^ at 28 days after implantation, while the mean tumor volumes of GL261-pirb increased by 100.35% compared with that of the GL261-nc group (Fig. 1D). Consistently, the mean tumor weight of GL261-pirb increased by 120.10% compared with that of GL261-nc (Figure S1F). As the immune microenvironment in the brain is different from that in other places, the function of pirb was further evaluated by survival analysis in an intracranial orthotopic tumor model. The survival time of the control group was significantly longer than that of the GL261-pirb^+^ group (Fig. 1E). All these results indicated that LILRB2/pirb is an oncogene that promotes GBM progression, resulting in a short survival time.

### The biological function of pirb in GBM

**Fig. 2:**
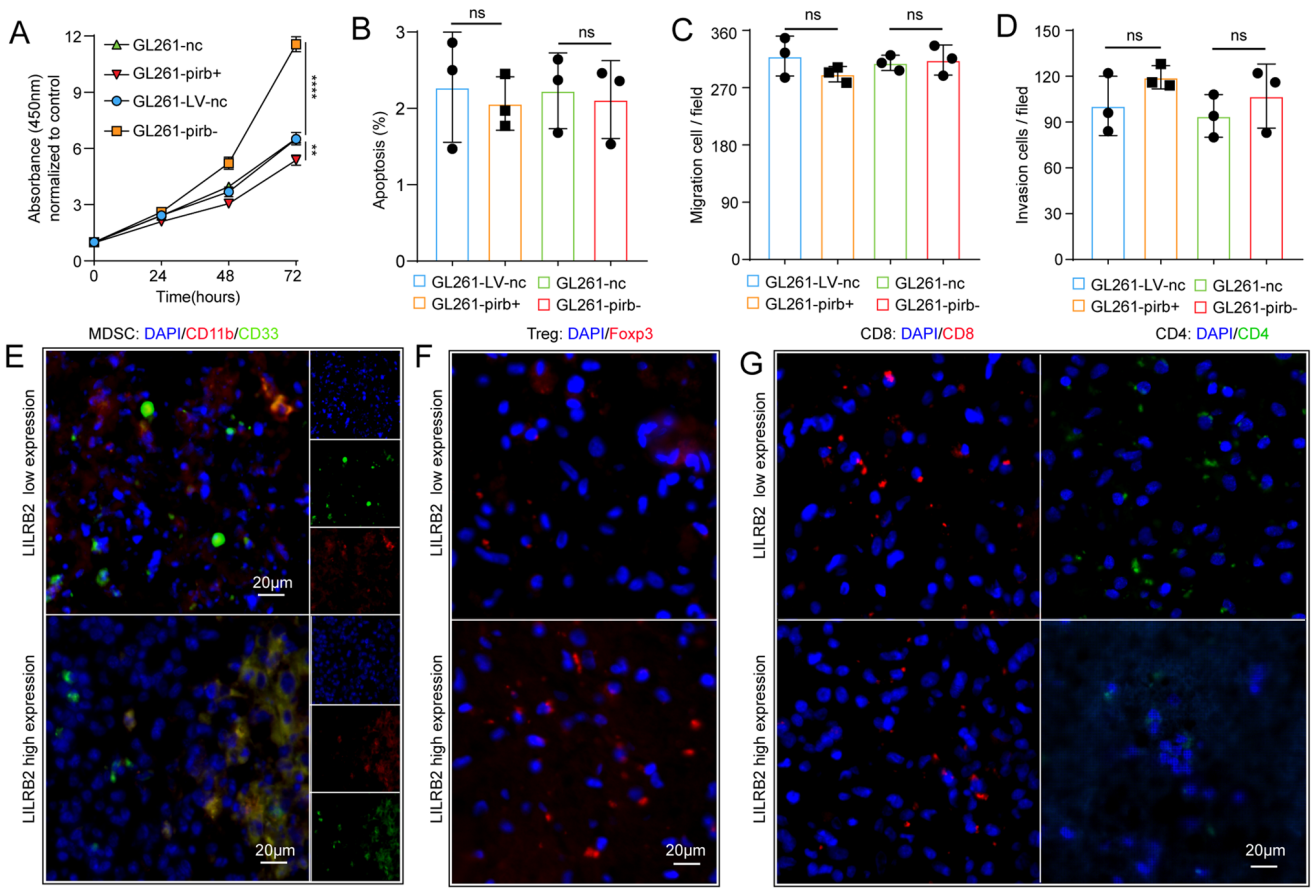
Pirb promotes GBM progression through an immunosuppressive TME. **A** The proliferation of GL261-LV-nc, GL261-pirb^+^, GL261-nc and GL261-pirb^−^ cells detected by CCK8 assays at 24, 48 and 72 h. Statistical analysis of cell apoptosis **B**, migration **C** and invasion **D** in GL261-LV-nc, GL261-pirb^+^, GL261-nc and GL261-pirb^−^ cells. The presence of MDSCs **E**, Tregs **F**, CD4 + and CD8 + T cells **G** in human GBM tissue detected by IF. *P < 0.05, ** P < 0.01, ***P < 0.001, or ****P < 0.0001, ns indicates no statistical significance

The biological function of pirb in GBM cells was evaluated. First, siRNA was used to downregulate pirb expression in GL261 cells and verified by WB (Figure S2A). Then, a CCK8 assay was conducted in GL261-LV-nc, GL261-pirb^+^, GL261-nc and GL261-pirb^−^ cells. The results of the CCK8 assay showed that the viability of GL261 cells was significantly inhibited by upregulating pirb and promoted by downregulating pirb (Fig. 2A), which was in contrast to the in vivo results. Afterward, cell apoptosis was measured by flow cytometry. We found that there was no significant difference in cell apoptosis when the expression of pirb in GL261 cells changed (Fig. 2B, Figure S2B). Next, the migration and invasion of GL261-LV-nc, GL261-pirb^+^, GL261-nc and GL261-pirb^−^ cells were evaluated. We found that there was no significant difference in migration and invasion (Fig. 2C-D, Figure S2C-D). Based on the paradoxical results presented, we speculated that pirb may function through immunity.

Previous studies have shown that LILRB2 functions as an immunosuppressive protein to induce T-cell senescence and suppress tumor immunity [30, 31]. CD8 + T cells, the main antitumor effector, CD4 + T cells, an important regulator, MDSCs, which play a central role in the regulation of immunosuppression, and Tregs, the main effector in the suppression of immunity, were selected and detected in GBM tissues. Thus, immunofluorescence staining (IF) was performed in human GBM tissues. Significantly high expression of CD11b and CD33 was observed in GBM tissues with high LILRB2 expression, indicating that higher LILRB2 expression in GBM tissue was related to more MDSCs (Fig. 2E). Subsequently, the expression of Foxp3 was detected, and the results indicated that more Treg cells were observed in GBM tissues with higher LILRB2 expression (Fig. 2F). In addition to MDSCs and Treg cells, T cells, including CD4 + T cells and CD8 + T cells, have an important role in tumor rejection [32]. Fewer infiltrated CD8 + T cells and CD4 + T cells were observed in human GBM tissues with high LILRB2 expression (Fig. 2G). Thus, LILRB2 is an immunosuppressive protein correlated with the immunosuppressive TME.

### Upregulated pirb expression leads to immunosuppression

**Fig. 3:**
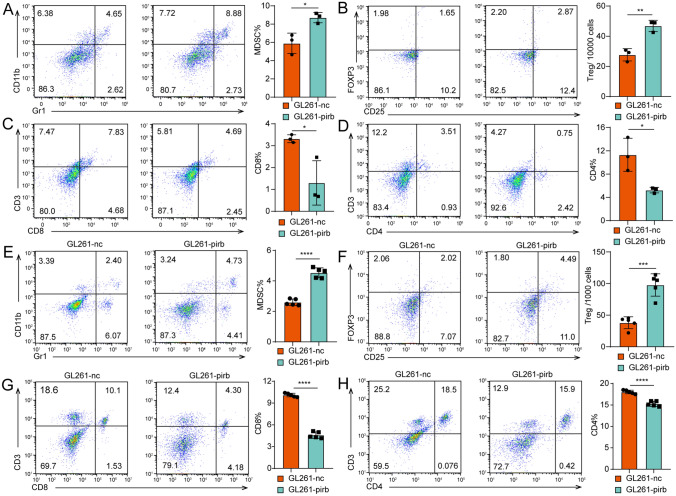
High pirb expression leads to immunosuppression. The percentages of MDSCs **A**, Tregs **B**, CD8 + T cells **C** and CD4 + cells **D** in the tumor sites detected by flow cytometry. The percentages of CD8 + T cells **E**, MDSCs **F**, CD4 + T cells **G** and Treg cells **H** in the spleens of tumor-bearing mice detected by flow cytometry. *P < 0.05, ** P < 0.01, ***P < 0.001, or ****P < 0.0001, ns indicates no statistical significance

As the previous studies indicated that LILRB2/pirb acted as a suppressor of immunity [30, 33, 34]. Thus, immunes cells were detected in the TME and circulation. The Tregs detected by flow cytometry were gated as shown in Figure S3A [35]. We found that more MDSCs were observed in GL261-pirb^+^ group than in GL261-LV-nc group (Fig. 3A). Consistently, more Tregs were detected in GL261-pirb^+^ group than in GL261-LV-nc group (Fig. 3B). We found more CD8 + and CD4 + T cells in the GL261-LV-nc group than in the GL261-pirb group (Fig. 3C, D). Next, the relationship between LILRB2 and immune cells in GL261-LV-nc and GL261-pirb^+^ orthotopic tumors was evaluated by IF. More MDSCs and Tregs in the GL261-pirb^+^ tumor sites, while fewer CD8 + T cells and CD4 + T cells were observed (Figure S3B).

The local antitumor immune response can be influenced by continuous communication of the periphery. Thus, a thorough understanding of immune responses to cancer must encompass immune cells (CD4 + T cells, CD8 + T cells, MDSCs and Tregs) across the peripheral immune system in addition to within the TME. The percentage of CD8 + T cells in the spleen of the GL261-pirb tumor-bearing mice decreased significantly compared to that in the GL261-nc tumor-bearing mice from 10.10% ± 0.25% to 4.49% ± 0.44% (*P* < 0.0001, Fig. 3E). In contrast, the percentage of MDSCs in the spleen of the GL261-pirb tumor-bearing mice was 4.54% ± 0.21%, which was significantly higher than the 2.60% ± 0.21% in the control group (*P* < 0.0001, Fig. 3F). Consistently, a significant increase in Tregs was detected in the spleen of the GL261-pirb tumor-bearing mice compared to the GL261-nc mice (Fig. 3G). Moreover, the percentage of CD4 + T cells in the GL261-pirb tumor-bearing mice was significantly lower than that in the GL261-nc tumor-bearing mice (Fig. 3H). These results indicated that pirb overexpression in GBM cells suppresses immunity by suppressing CD8 + T cells and inducing MDSCs in the TME and circulation.

### Pirb can be secreted via sEVs

**Fig. 4:**
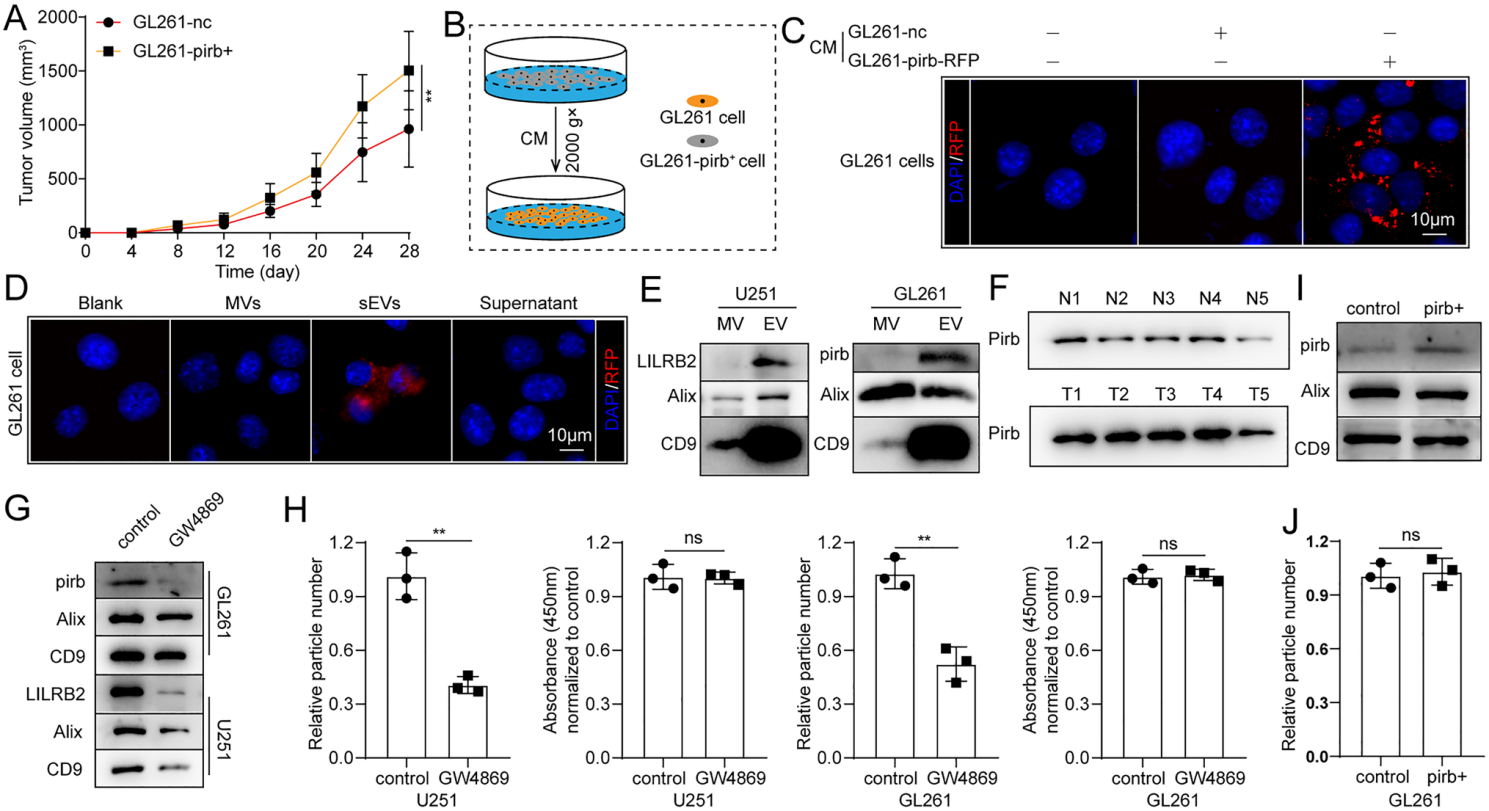
Pirb can be secreted via sEVs. **A** The growth curve of subcutaneous tumors. **B** The schematic diagram. **C** RFP fluorescence in GL261 cells in transwell plates. **D** RFP fluorescence in GL261 cells after incubation with CM from GL261-pirb cells with different treatments (MVs, sEVs and supernatant). **E** pirb/LILRB2, Alix and CD9 in U251-MVs, U251-sEVs, GL261-MVs and GL261-sEVs detected by WB. **F** pirb expression in sEVs from blood samples of the control mice and the tumor-bearing mice detected by WB. **G** LILRB2/pirb, Alix, and CD9 in U251-sEVs and GL261-sEVs detected by WB before or after GW4869 treatment. **H** The sEV amount and cell number detected by nanoflow cytometry and CCK8 assays. **I** pirb, Alix and CD9 in GL261-sEVs and GL261-pirb-sEVs were detected by WB. **J** The amount of sEVs in GL261 and GL261-pirb cells was detected by nanoflow cytometry. *P < 0.05, ** P < 0.01, ***P < 0.001, or ****P < 0.0001, ns indicates no statistical significance

Accidently, we found that the tumor progression of GL26-nc can be affected by GL261-pirb^+^ in the same mouse. Briefly, a subcutaneous tumor model was constructed using both sides of C57BL/C mice with GL261-nc and GL261-pirb^+^, as shown in Figure S4A-B. Statistical analysis showed that the GL261-nc tumor volume in Fig. 1C was 358.85 ± 168.58 mm^3^, while the GL261-nc tumor volume in Fig. 4A was 962.40 ± 353.43 mm^3^ (*P* = 0.0087), which implied that pirb in G261-pirb regulates the function of GL261-nc in some way. Because of the tumor-derived factors, immunity in GBM patients was substantially suppressed. Current studies have demonstrated that tumor cell-derived sEVs transmit oncoproteins between tumor cells, stromal cells and immune cells to regulate the function of recipient cells, promoting tumor progression and suppressing the immune system [36, 37]. Thus, we speculated that pirb can be secreted by GBM cells and regulate the function of immune cells.

Thus, GL261-pirb-RFP (overexpressed with red fluorescence protein, RFP) was constructed, and the overexpression of pirb in GL261 cells was confirmed by WB (Figure S4B). Currently, study have shown that extracellular vesicle can be mainly divided into small extracellular vesicle and macrovesicle [38]. After that, the conditioned medium (CM) of GL261-nc and GL261-pirb-RFP cells was harvested and cultured with GL261 cells after removal of cell debris, as shown in the schematic diagram in Fig. 4B. RFP fluorescence was observed in GL261 cells cultured with CM from GL261-pirb-RFP cells but not in GL261-nc cells (Fig. 4C). Then, the components of the CM were isolated by differential centrifugation and cultured with GL261 cells to determine the way in which pirb was transferred. We found that there was no RFP fluorescence in GL261 cells after incubation with MVs from GL261-pirb-RFP (GL261-pirb-RFP-MV) or supernatant (without MVs and sEVs) from GL261-pirb-RFP, while RFP fluorescence was observed in GL261 cells cultured with sEVs from GL261-pirb-RFP (GL261-pirb-RFP-sEVs) (Fig. 4D). Therefore, we concluded that pirb can be secreted by tumor cells and carried by sEVs.

Furthermore, sEVs from U251, U87 and GL261 cells were isolated and verified according to the guidelines (Figure S4D-F). The presence of pirb in GL261-sEVs and LILRB2 in U251-sEVs was detected by WB but not in the MVs of GL261 or U251 cells (Fig. 4E). The presence of LILRB2 in sEVs was also confirmed by the result of U87 cells (Figure S4G). In addition, sEVs from the blood of the tumor-bearing mice and the control mice were isolated, and sEVs secreted by GL261-pirb tumors contained more pirb than sEVs from GL261-nc tumors (Fig. 4F). Finally, we found that the secretion of pirb was inhibited by GW4869, a kind of medicine used to inhibit the secretion of sEVs [39], both in U251 and GL261 cells (Fig. 4G). The sEV amount and cell number were detected by nanoflow cytometry and CCK8 assays, respectively. We found that the secretion of sEVs in GL261 and U251 cells was inhibited after incubation with GW4869 (10 μM) (Fig. 4H). Moreover, sEVs from the same volume of CM of GL261-nc and GL261-pirb were isolated. We found that more pirb was secreted by GL261-pirb cells than by GL261-nc cells, and the increase in pirb was independent of the secretion rate of sEVs (Fig. 4I-J). These results suggest that LILRB2/pirb can be transmitted between cells via sEVs.

### sEV-pirb-induced MDSCs in the circulation contribute to the formation of an immunosuppressive TEM

**Fig. 5:**
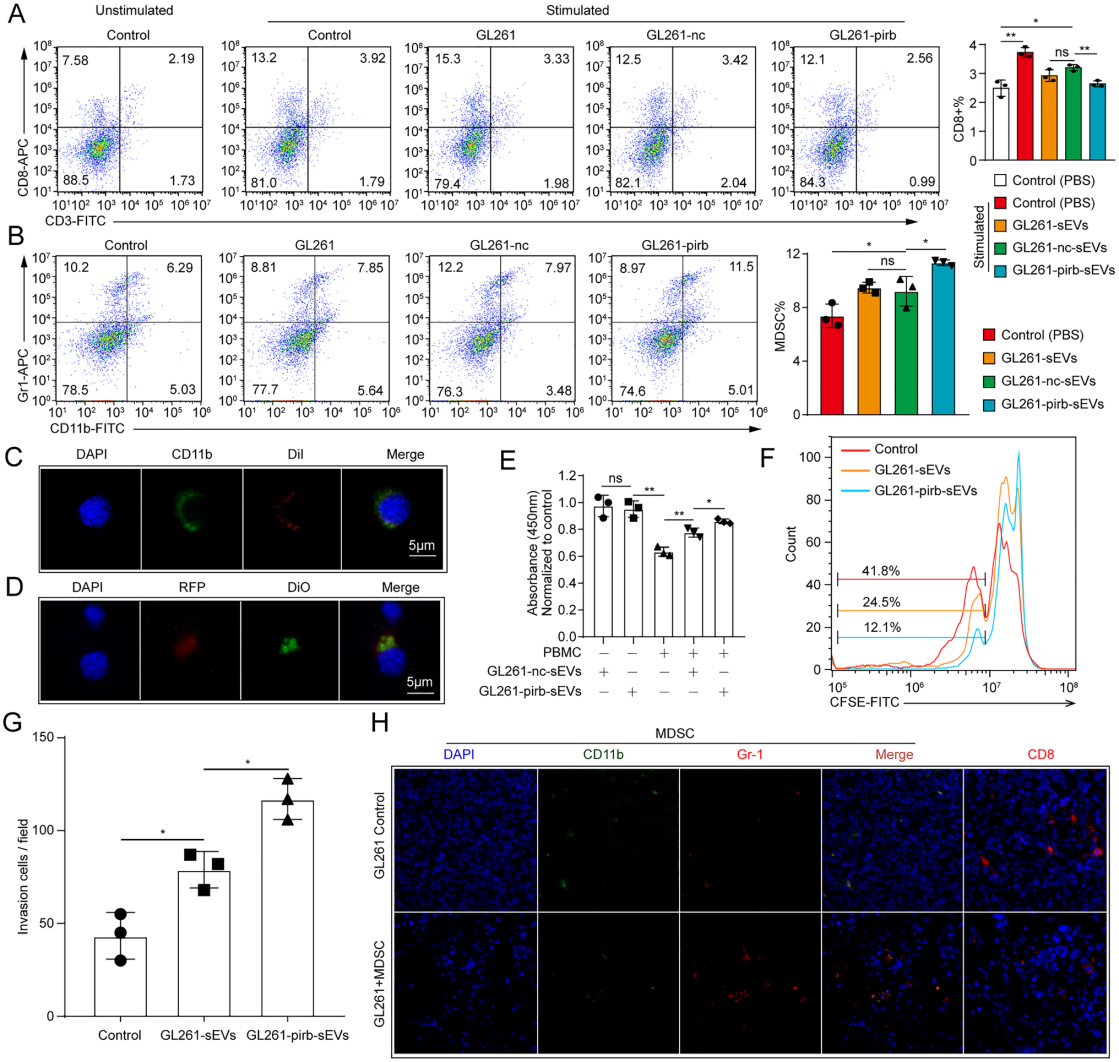
sEVs-pirb inhibit immunity by promoting MDSC formation. **A** The percentage of CD8 + T cells in spleen cells after treatment with GL261-sEVs, GL261-nc-sEVs or GL261-pirb-sEVs was detected by flow cytometry. **B** The induction of MDSCs by GL261-sEVs, GL261-nc-sEVs or GL261-pirb-sEVs was detected by flow cytometry. **C** The interaction of GL261-sEVs with myelin cells was detected by IF. Scale bar: 10 μm. **D** The presence of GL261-sEVs-pirb on the membrane of myelin cells was detected by IF. Scale bar: 10 μm. **E** The tumor cell killing assay performed in GL261 cells by CCK8 assays. **F** Histogram of CFSE in CD8 + T cells after incubation with MDSCs induced by GL261-sEVs or GL261-pirb-sEVs. **G** A schematic diagram of the MDSC invasion assay. **H** MDSCs in GL261 tumors with or without MDSC treatment detected by IF. *P < 0.05, ** P < 0.01, ***P < 0.001, or ****P < 0.0001, ns indicates no statistical significance

Previous studies have shown that the function of immunity can be regulated by proteins on sEVs [40]. Then, flow cytometry was performed to evaluate the function of pirb on sEVs after spleen cells were treated with GL261-nc-sEVs or GL261-pirb-sEVs. We found that the percentage of CD8 + T cells was downregulated by GL261-nc-sEVs, while the immunosuppressive ability of G261-pirb-sEVs was stronger than that of GL261-sEVs (Fig. 5A). The association between LILRB2 and MDSCs was evaluated by bioinformatics analysis on the GEPIA website. CD33, ITGAM, S100A8 and S100A9 were selected as gene signatures of MDSCs [41]. We found a positive correlation between LILRB2 and CD33 (R = 0.82)/ITGAM (R = 0.75)/S100A8 (R = 0.67)/S100A9 (R = 0.69) (Figure S5A). Furthermore, the formation and expansion of MDSCs were activated by GL261-sEVs and GL261-pirb-sEVs. We found that the immunosuppressive ability of GL261-pirb-sEVs was stronger than that of GL261-sEVs (Fig. 5B). Then, confocal microscopy analysis showed that the fluorescence of DiI merged with the fluorescence of CD11b, indicating that GL261-sEVs may interact with the membrane of the splenocyte in some way or internalized by recipient cells (Fig. 5C). The presence of RFP and DiO fluorescence signal in splenocytes indicated the interaction between sEVs-pirb with splenocytes or the internalization of sEVs-pirb by splenocytes (Fig. 5D). The function of sEVs-pirb was further analyzed by a tumor cell killing assay (Fig. 5E). We found that the antitumor effect of CD8 + T cells was inhibited by GL261-sEVs and GL261-pirb-sEVs, but the immunosuppressive ability of GL261-pirb-sEVs was significantly stronger than that of GL261-sEVs. Moreover, the proliferation of CD8 + T cells was detected in the presence of MDSCs induced by GL261-sEVs or GL261-pirb-sEVs. As shown in Fig. 5F, the proliferation of CD8 + T cells was inhibited under the suppression of MDSCs induced by GL261-pirb-sEVs, which is consistent with previous results.

Next, the migration of MDSCs was evaluated in a transwell plate in the presence of GL261-sEVs or GL261-pirb-sEVs. We found that the migration of MDSCs was promoted by GL261-pirb-sEVs compared to GL261-sEVs (Fig. 5G, Figure S5B). Furthermore, MDSCs induced by GL261-pirb-sEVs were isolated, and these MDSCs were intravenously injected into the GL261 tumor-bearing mice, as shown in the schematic diagram (Figure S5C). The tumor samples were harvested 3 days after the injection. Then, IF was performed to detect MDSCs in the tumor samples. We found more MDSCs present in the tumor sites than in the controls after the injection of MDSCs (Fig. 5H). Therefore, we believe that sEV-pirb-induced MDSCs in the circulation can be a source of MDSCs in the TME. All these results indicated that sEV-pirb can be an immunosuppressor to suppress antitumor immunity by forming an immunosuppressive TME.

### sEVs-pirb facilitate the progression of GBM

**Fig. 6:**
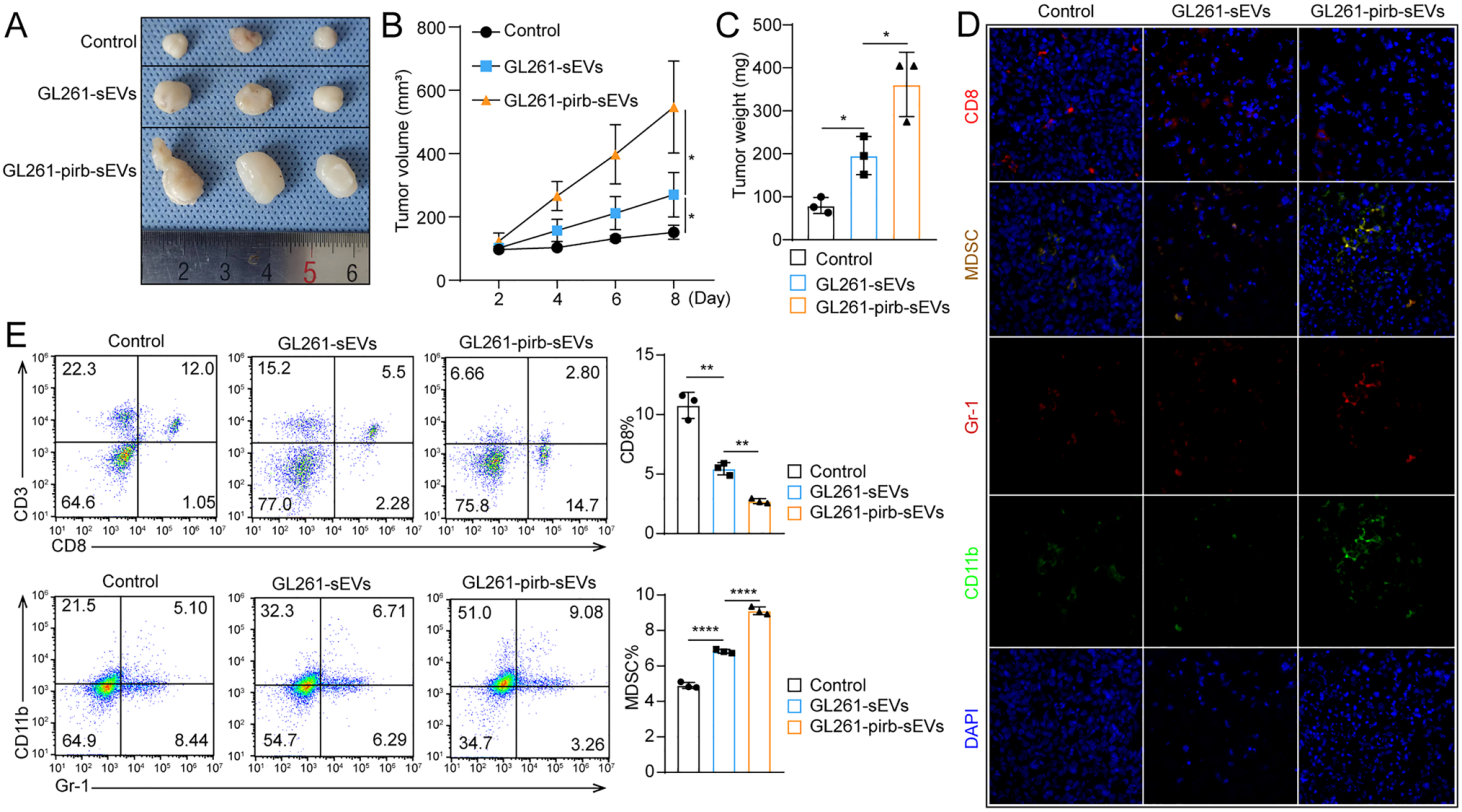
sEVs-pirb facilitate the progression of GBM. **A** Image of the subcutaneous tumor models (*n* = 3). **B** The growth curve of the subcutaneous tumor models. **C** The statistical analysis of the tumor weight of GL261-nc and GL261-pirb. **D** CD8 + T cells and MDSCs in the tumor site detected by IF after treatment with GL261-sEVs or GL261-pirb-sEVs. **E** The percentage of CD8 + T cells and MDSCs in the spleen of tumor-bearing mice detected by flow cytometry; the statistical analysis is shown on the right

To verify the function of sEVs-pirb in vivo, we generated subcutaneous tumor models in C57BL/6 mice with GL261 cells (Fig. 6A). Injection of GL261-nc-sEVs (1 × 10^10^ particles/mL, 100 μL) significantly promoted the progression of GBM compared with that of the control group (control vs. GL261-nc-sEVs: 151.21 ± 22.45 mm^3^ vs. 270.02 ± 70.62 mm^3^, *P* = 0.05), while the protumor ability of GL261-pirb-sEVs was significantly stronger than that of GL261-sEVs (GL261-nc-sEVs vs. GL261-pirb-sEVs: 270.02 ± 70.62 mm^3^ vs. 547.08 ± 145.49 mm^3^, *P* = 0.0413) (Fig. 6B). Consistently, the mean tumor weight of GL261-sEVs increased 94.32% compared with that of the control, and the mean tumor weight of GL261-pirb increased by 84.49% compared with that of GL261-sEVs (Fig. 6C).

In addition, tumor samples were collected, and a significant decrease in CD8 + T cells and an increase in MDSCs were observed in the tumor sites treated with GL261-sEVs, while fewer tumor-infiltrating CD8 + T lymphocytes and more MDSCs were observed in the tumor sites treated with GL261-pirb-sEVs than in those treated with GL261-sEVs (Fig. 6D). Accordingly, the immune cells in the spleen of the tumor-bearing mice were also detected by flow cytometry. As shown in Fig. 6E, the percentage of CD8 + T cells in the control group was 10.78% ± 1.1%, decreased to 5.45 ± 0.52 in the GL261-sEV group, and decreased to 2.73% ± 0.21%. While the percentage of MDSCs in the control was 4.90% ± 0.17%, the percentage of MDSCs reached 6.82% ± 0.11% and 9.11% ± 0.22% in GL261-sEVs and GL261-pirb-sEVs, respectively. We found that pirb on sEVs can exert its function through the immune system by inducing MDSCs.

## Discussion

Immunotherapy is a promising treatment for tumors that exert antitumor functions by reactivating the suppressed CD8 + T cells in the TME. However, the benefit of immunotherapy for GBM is limited because of the immunosuppressive TME in GBM patients [42, 43]. To enhance the therapeutic effect of immunotherapy, researchers need to elucidate the underlying mechanism by which the immunosuppressive TME is formed. In the present study, we found that pirb can be secreted via sEVs to induce the formation of MDSCs, and the increased MDSCs in circulation infiltrate into the TME of GBM, which forms an immunosuppressive TME, promoting the progression of GBM and leading to a poor prognosis of GBM patients.

LILRB2, also named ILT4, monocyte/macrophage immunoglobulin-like receptor 10 (MIR-10), or CD85d, consists of four extracellular tandem Ig-like domains, a transmembrane region and a cytoplasmic tail with three immunoreceptor tyrosine inhibitory motifs (ITIMs), which are mainly expressed in innate immune cells. Recently, studies have shown that LILRB2 is also enriched in tumor cells [30, 44]. Research has shown that tumor-derived ILT4 is involved in the induction of cell senescence in naïve/effector T cells by activating the MAPK ERK1/2 signaling pathway [30]. Another study showed that overexpressed ILT4 in non-small cell lung cancer cells recruits tumor-associated macrophages and induces M2-like polarization. However, the underlying mechanism by which ILT4 in tumor cells regulates immune cells is still unknown. The immunosuppressive ability of LILRB2 was also confirmed in our study. Furthermore, we isolated different components of the GL261 culture medium and found that LILRB2 in GBM cells can be delivered through sEVs to other cells, such as tumor cells and immune cells. The secreted sEVs-LILRB2 interacted with immune cells directly, inducing the formation of MDSCs.

Because of the importance of MDSCs in the balance of immunity, the formation of MDSCs is strictly regulated in the human body. Otherwise, the immune system will be overactive in healthy humans when MDSC formation is disordered. However, the regulatory mechanisms are hijacked by tumor cells to create an immunosuppressive environment. Here, our results demonstrated that sEVs-LILRB2/pirb are regulators of the formation of MDSCs. Furthermore, studies have shown that programmed cell death ligand 1 (PDL1) on sEVs is a pool that directly interacts with programmed cell death 1 on cells to promote tumor growth [45] and mediate immune evasion in GBM [46], which affects the outcome of immunotherapy. Thus, we propose that the existence of sEVs-LILRB2 in circulation also needs to be considered.

Although GBM-sEVs-pirb have been demonstrated to suppress immunity, the detailed mechanism still needs to be explored. Because MDSCs consist of a group of heterogeneous cells, the induced subgroup needs to be investigated, and the mechanism through which pirb induces the formation of MDSCs is still unknown.

Overall, this study indicates that sEVs-pirb induce MDSCs and facilitate the formation of immunosuppression both in the system and microenvironment. The secreted sEVs-pirb may act as a pool to bind monoclonal antibodies, resulting in resistance to immunotherapy. Therefore, blocking LILRB2 in GBM patients may be a potential way to disrupt the formation of MDSCs, which may be a therapeutic target to enhance the outcome of immunotherapy.

## Conclusion

Immunotherapy is an important treatment for tumors, but the immunosuppressive TME of GBM is the major hindrance preventing GBM patients from benefitting from it. Therefore, exploration of the formation of an immunosuppressive GBM TME is important. In this study, we found that pirb can be secreted by sEVs and induce MDSCs by direct interaction with myelin cells, suppressing the immune system. The increased MDSCs may infiltrate the TME, forming an immunosuppressive TME. Our research provides an explanation for the formation of an immunosuppressive TME, which can be a therapeutic target.

### Supplementary Information

Below is the link to the electronic supplementary material.

Supplementary file1 (DOCX 3681 kb)

## Data Availability

The datasets generated during and/or analyzed during the current study are available from the corresponding author on reasonable request.
