# Supplementary material for: LILRB2-containing small extracellular vesicles from glioblastoma promote tumor progression by promoting the formation and expansion of myeloid-derived suppressor cells
Source: Cancer Immunol Immunother. 2023 Feb 28;72(7):2179–93. doi: 10.1007/s00262-023-03395-6 (PMC10264499; doi:10.1007/s00262-023-03395-6)
Supplement: Supplementary file 1 — Supplementary file1 (DOCX 3681 kb) [file 262_2023_3395_MOESM1_ESM.docx]

Supplementary information

LILRB2-containing small extracellular vesicles from glioblastoma promote tumor progression by promoting the formation and expansion of myeloid-derived suppressor cells


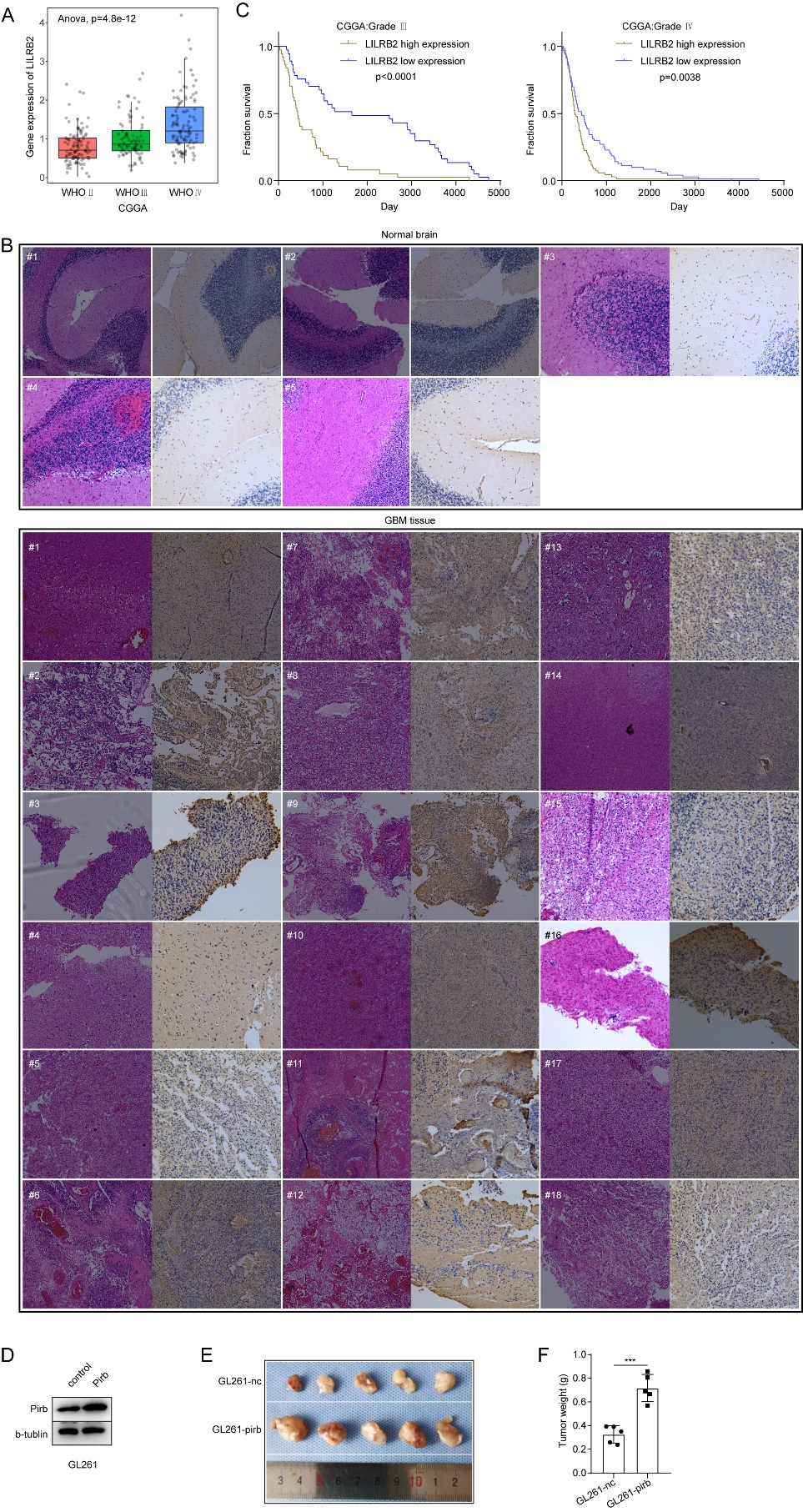


**Figure S1:** (A) Expression of LILRB2 mRNA in glioma samples (Grade Ⅱ, n =103), glioma samples (Grade Ⅲ, n =79) and glioma samples (Grade Ⅳ, n = 139). (B) Kaplan-Meier analysis of overall survival and disease-free survival of high grade glioma (Grade Ⅲ and Grade Ⅳ) patients showing high LILRB2 expression (50% cutoff) and low LILRB2 expression (50% cutoff). (C) The representative images of normal brain tissues (n=5) and GBM tissues (n=18) detected by IHC and HE. (D) The expression of pirb in GL261-nc and GL261-pirb+ detected by WB. (E) Image of subcutaneous tumors of GL261-nc (n=5) and GL261-pirb (n=5). (F) The statistical analysis of the tumor weight of GL261-nc and GL261-pirb.


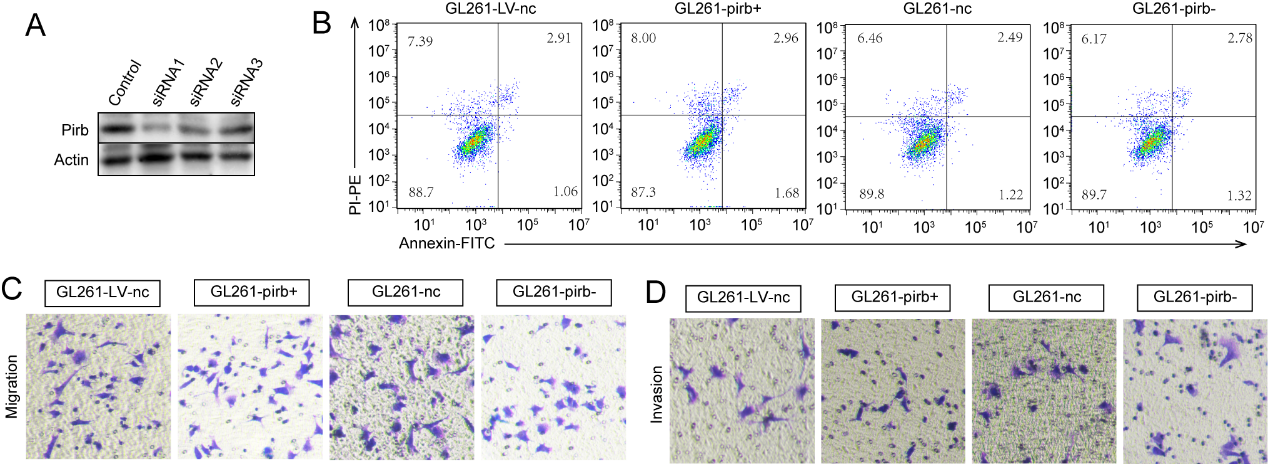


**Figure S2**: (A) The expression of pirb of control and GL261-pirb- detected by WB. (B) Representative images of cell apoptosis detected by flow cytometry. Representative images of cell migration (C) and invasion (D) conducted in transwell plants.


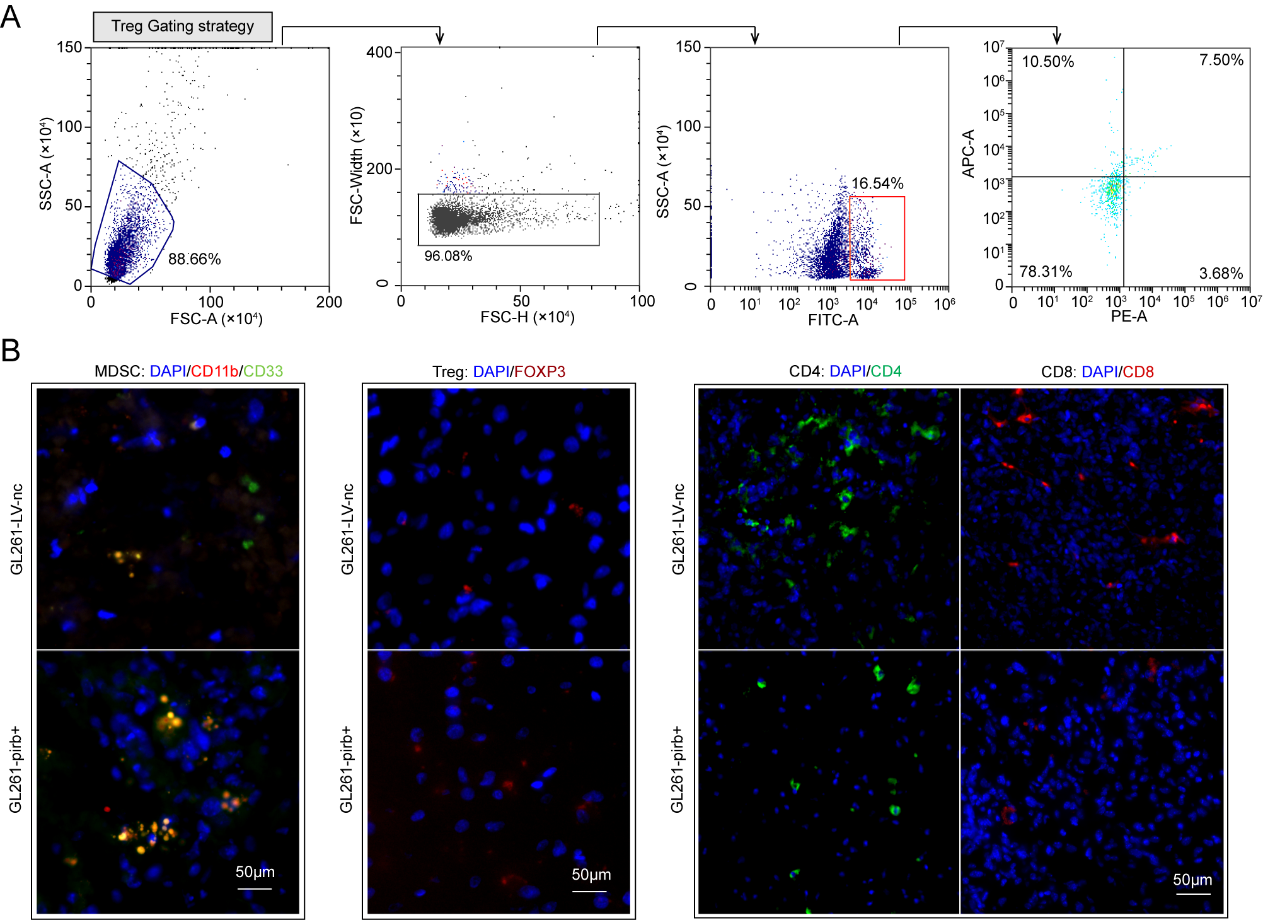


**Figure S3**: (A) The gating strategy of the Treg. (B) Correlation between pirb expression with MDSC, Treg, CD8+ T cell and CD4+ T cell.


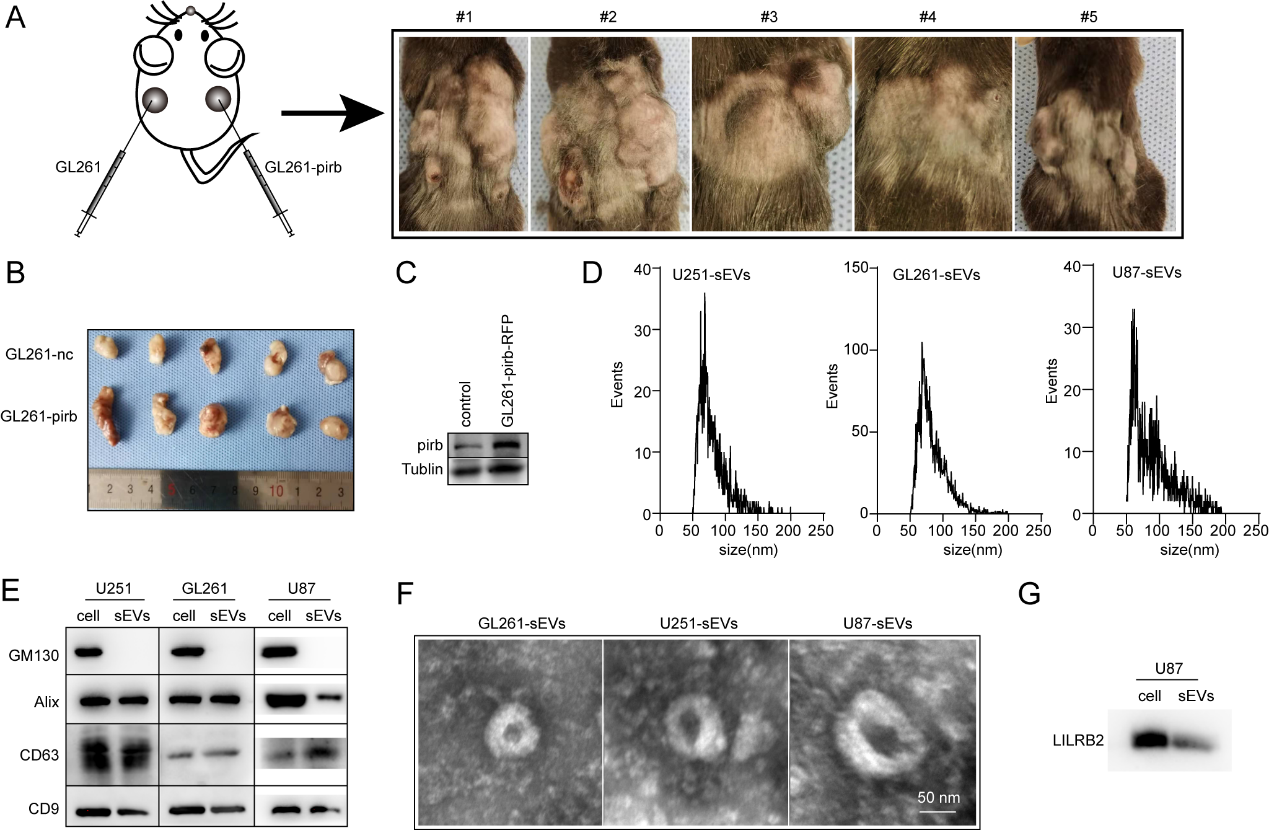


**Figure S4:** (A-B) The schematic diagram of mice tumor model (A) and the images of subcutaneous tumor in vivo of C57BL/C mice on both sides. (C) the expression of pirb in control group and GL261-pirb-RFP group detected by WB. (D) The size distribution of U251-sEVs, U87-sEVs and GL261-sEVs detected by nanoflow cytometry. (E) GM130, CD63, CD9 and Alix of U251 cells, U251-sEVs, U87 cells, U87-sEVs, GL261 cells and GL261-sEVs detected by WB. (F) The morphologies of U251-sEVs, U87-sEVs and GL261-sEVs detected by TEM. (G) the expression of LILRB2 in U87 cells and U87-sEVs detected by WB.


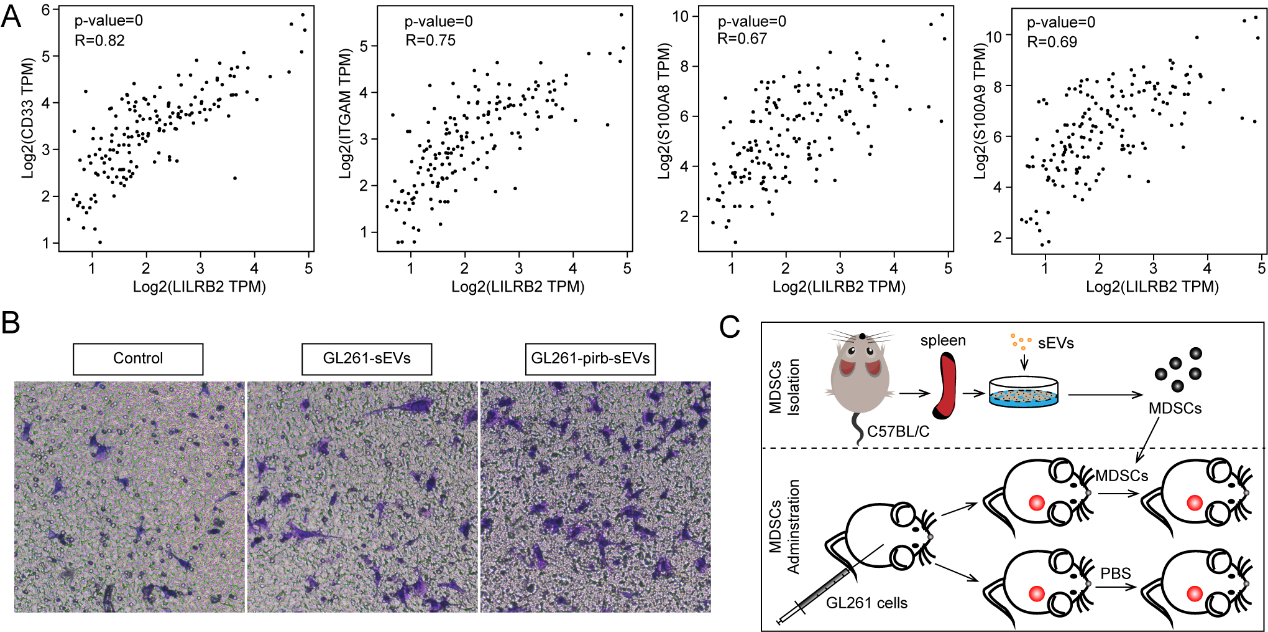


**Figure S5**: (A) The association between LILRB2 and CD33, ITGAM, S100A8, S100A9 analyzed in GEPIA website. (B)The schematic diagram of mice tumor model. (B) The representative images of migration assay. The migration ability of MDSCs was detected in a transwell plant after the administration of GL261-sEVs or GL261-pirb-sEVs.

Supplementary materials and methods

HE staining

The GBM tissue and normal brain tissues were rapidly isolated and fixed with 4% paraformaldehyde in PBS overnight at 4℃ and embedded in paraffin. The fixed brains were sectioned into slices with thicknesses of 20 μm. The samples were then stained with hematoxylin and eosin and imaged by DM6 microscopy (LEICA, Germany).
